# Satisfaction of Emergency Physicians with the Care Provided to Patients with Cardiovascular Diseases in the Northern Region of Minas Gerais

**DOI:** 10.5935/abc.20180143

**Published:** 2018-08

**Authors:** Milena Soriano Marcolino, João Antonio de Queiroz Oliveira, Grace Kelly Matos e Silva, Thatiane Dantas Dias, Barbara Campos Abreu Marino, André Pires Antunes, Antonio Luiz Ribeiro, Clareci Silva Cardoso

**Affiliations:** 1 Telehealth Center, University Hospital, Universidade Federal de Minas Gerais (UFMG), Belo Horizonte, MG - Brazil; 2 Medical School, Universidade Federal de Minas Gerais, Belo Horizonte, MG - Brazil; 3 Universidade Estadual de Montes Claros, Montes Claros, MG – Brazil; 4 Universidade Federal de São João del-Rei, Divinópolis, MG – Brazil

**Keywords:** Cardiovascular Diseases, Myocardial Infartion, Acute Coronary Syndrome, Epidemiology, Health Profile, Quality Indicators, Health Care, Emergency Medical Services

## Abstract

**Background:**

The dissatisfaction of health professionals in emergency services has a negative influence on both the quality of care provided for acute myocardial infarction (AMI) patients and the retention of those professionals.

**Objective:**

To assess physicians’ satisfaction with the structure of care and diagnosis at the emergency services in the Northern Region of Minas Gerais before the implementation of the AMI system of care.

**Methods:**

This cross-sectional study included physicians from the emergency units of the ambulance service (SAMU) and level II, III and IV regional hospitals. Satisfaction was assessed by using the CARDIOSATIS-Team scale. The median score for each item, the overall scale and the domains were calculated and then compared by groups using the non-parametric Mann-Whitney test. Correlation between time since graduation and satisfaction level was assessed using Spearman correlation. A p value < 0.05 was considered significant.

**Results:**

Of the 137 physicians included in the study, 46% worked at SAMU. Most of the interviewees showed overall dissatisfaction with the structure of care, and the median score for the overall scale was 2.0 [interquartile range (IQR) 2.0-4.0]. Most SAMU physicians expressed their dissatisfaction with the care provided (54%), the structure for managing cardiovascular diseases (52%), and the technology available for diagnosis (54%). The evaluation of the overall satisfaction evidenced that the dissatisfaction of SAMU physicians was lower when compared to that of hospital emergency physicians. Level III/IV hospital physicians expressed greater overall satisfaction when compared to level II hospital physicians.

**Conclusion:**

This study showed the overall dissatisfaction of the emergency physicians in the region assessed with the structure of care for cardiovascular emergencies.

## Introduction

The recent decades have witnessed a significant reduction in mortality from cardiovascular diseases resulting from the advances in primary prevention and treatment of acute coronary syndrome.^1^^-^^4^ Despite being a worldwide trend, it is more evident in developed countries, where proper and timely treatment is available.^5^ The “S*istema de Informação de Mortalidade* (SIM) of the *Ministério da Saúde”* (Brazilian Health Ministry Mortality Information System (SIM)) recorded, in 2015, approximately 350 000 deaths from cardiovascular diseases, which, in Brazil, remain the leading cause of proportional mortality, accounting for 27.6% of the deaths in 2015. Additionally, it is the major cause of years of life lost due to premature death.^6^

Of the cardiovascular diseases, acute myocardial infarction (AMI) is the most frequent cause of death (26.0%),^6^ and mortality at public healthcare services is higher than at private healthcare services.^7^ That difference may be attributed to difficulties experienced by AMI patients to have access to intensive care, reperfusion methods and the therapeutic measures established for AMI.^7^^,^^8^ Such difficulties can have a negative impact on the satisfaction of emergency healthcare professionals, which might impact negatively the retention of those professionals in regions lacking healthcare structure. The current crisis in emergency services is well known.^9^ Thus, assessing the factors related to it, such as the satisfaction of healthcare professionals with healthcare structure, is paramount.

The Northern Region of Minas Gerais comprises 89 municipalities, occupying an area of approximately 128 000 km^2^, with around 1 594 000 inhabitants. That region differs from the rest of the Minas Gerais state, as it has a human development index close to those of the poorest states in Northeastern Brazil.^10^ Similar to the rest of Brazil, specialized healthcare is concentrated in the largest municipality of the region, Montes Claros, and mortality from AMI is very high,^11^ motivating the implementation of a project to organize the AMI system of care in the region.

This study aimed at assessing the satisfaction of physicians with the structure of care and diagnosis of public emergency services in the Northern Region of Minas Gerais before the implementation of the AMI system of care in the region.

## Methods

### Organization of the Care Network for Emergency Services in the Northern Region of Minas Gerais

The care network for emergency services in the Northern Region of Minas Gerais is an integrated network that comprises a regional mobile emergency care service (SAMU, in Portuguese), and micro- and macroregional hospitals. The *“Projeto Estadual de Redes de Atenção”* has categorized the hospitals according to their expertise and their response to two major problems that impact the potential years of life lost: severe trauma and cardiovascular and cerebrovascular emergencies.^12^

SAMU has a macroregional scope, attending 86 of the 89 municipalities of the region, with 7 advanced ambulances (with ambulance driver, nurse and physician), 40 basic ambulances (with an ambulance driver and two nursing technicians) and a rapid interception vehicle. There is only one regulatory center.

The regional hospitals are as follows:

- Level I hospitals: provide several “high-complexity” procedures, such as neurosurgery, vascular surgery and interventional angiography, resuscitation room (red) with mobile radiography and ultrasound, computerized tomography, operating rooms for complex surgeries, heliport with exclusive access, trauma surgical team, transfusion unit, and several differentiated and special hospital beds at intensive care and coronary care units.
- Level II hospitals: located in municipalities with more than 200 000 inhabitants, similar to level I hospitals, except for the absence of angiography, vascular surgery and coronary care units.
- Level III hospitals: located in municipalities with more than 100 000 inhabitants, destined to patients’ stabilization until definite transfer to a level I or level II hospital. Their minimum requirements are: emergency healthcare professionals, general surgery, radiology, anesthesiology, transfusion unit and general intensive care unit.
- Level IV hospitals: located in areas that lack healthcare, which are more than 60 minutes away from a reference microregional hospital.^12^^,^^13^

### Implementation of the AMI System of Care in the Northern Region of Minas Gerais: Minas Telecardio II Project

*Minas Telecardio II Project* was aimed at implementing and assessing the AMI System of Care in the Northern Region of Minas Gerais and at evaluating its impact on AMI mortality. It was a quasi-experimental study conducted from June 19, 2013 to May 19, 2015 in three steps: (i) establishment of the baseline; (ii) implementation of the AMI Sysem of Care with the mobile tele-electrocardiology system and the new operational flow, in addition to training healthcare professionals of the pre-hospital and hospital emergency services of the region; and (iii) reassessment of the quality indicators for the care provided after the implementation. All phases have been concluded and detailed previously.^14^

The satisfaction of the group of physicians with the structure of care provided to patients with cardiovascular diseases was one of the aspects assessed in the study baseline, being the object of this article.

### Study design and satisfaction assessment

This is a cross-sectional study. Emergency physicians from SAMU and from the level II, III and IV regional hospitals that comprise the emergency network of the Northern Region of Minas Gerais participated in this study. The eligibility criteria were as follows: i) be a regular registered member at the Regional Council of Medicine; ii) provide care at SAMU and/or emergency centers of Northern Region of Minas Gerais’ regional hospitals.

The research team visited all advanced ambulances of SAMU in the region. Due to the long distance between the regional hospitals, which would hinder the evaluation of the physicians’ satisfaction in all of them, a random selection was performed by use of probabilistic simple random sampling. Thus, a numerical list was created, and the municipalities were selected, so that there would be one level III or IV hospital per microregion in the sample. Two level III hospitals and five level IV hospitals were selected.

Assessment of the physicians’ satisfaction was performed with the CARDIOSATIS-Team scale, specifically developed to evaluate physicians’ satisfaction with the care provided to cardiovascular emergencies. It follows the international standards for the creation of tools and has good validity and reliability for the Brazilian context.^15^^-^^17^ It is a self-administered tool with 11 closed items and 3 open questions. The open questions include information on access to and interest in professional qualification. The closed items include overall satisfaction and two domains: i) *satisfaction with the care provided;* and ii) *satisfaction with the structure of care and diagnosis*. Each item is assessed by use of a five-point Likert scale, where a score of 4 or 5 indicates higher satisfaction, a score of 1 or 2 indicates dissatisfaction, and a score of 3 indicates average satisfaction with the item assessed (‘neither’).

Each participant received a questionnaire with the scale and filled it out individually, after providing written informed consent. Those procedures were supervised by a previously trained team, which was available for clarifications, checking the professionals’ understanding and answering all their doubts.

### Statistical analysis

The statistical analysis was performed by using the IBM SPSS software, version 19.0 (IBM Corp, Armonk, NY). Categorical variables were described as absolute and relative frequency, and continuous variables as measures of central trend and dispersion [median and interquartile range (IQR)]. Data distribution was not normal, as assessed by use of the Kolmogorov-Smirnov test, thus, nonparametric tests were used. The statistical analysis was performed for groups (SAMU *versus* non-SAMU) and non-SAMU subgroups (level II hospitals *versus* level III/IV hospitals). Categorical variables were compared by using the chi-square test. The median score for each item, overall scale and domains were calculated and compared by using the nonparametric Mann-Whitney U test to assess the existence of difference, and a 5% significance level was used. The correlation between professional training time and overall satisfaction was assessed by use of Spearman correlation (r_s_).

### Ethical aspects

This study was approved by the Ethics Committee of Research of the Universidade Federal de Minas Gerais, number 260/09, aligned with the resolution CNS 466/12. All physicians provided written informed consent to participate in the study.

## Results

Of the 164 professionals, 137 (83.5%) completed the questionnaire. Of the respondents, 63 (46.0%) provided care at SAMU emergency units, and 74 (54.0%), at hospital emergency services. Among these, 28 (37.8%) worked at level II hospitals, and 46 (62.2%), at level III/IV hospitals.

Table 1 shows the descriptive characteristics of the groups. The median number of years since graduation was 5.3 (IQR 1.8-12.7), and it was similar when comparing physicians working at the SAMU emergency units and those at the hospital emergency services, except for those working at level III/IV hospitals. Most physicians were male (67.9%) and specialized (68.6%), and that proportion was higher at level III/IV hospitals when compared to the proportion of specialists at level II hospitals and SAMU units. The most common medical specialties were internal medicine (29.1%), pediatrics (9.5%), surgery (7.2%) and gynecology and obstetrics (7.2%). No statistically significant difference was observed between the groups regarding the distribution in the different specialties (SAMU vs non-SAMU, p = 0.168; level II hospitals vs level III/IV hospitals, p = 0.214).

**Table 1:** Distribution of the physicians according to time since graduation, sex and specialty

Characteristics	Overall total (n = 137)	Non-SAMU (n = 74)			SAMU (n = 63)
		Level II hospitals (n = 28)	Level III/IV hospitals (n = 46)	Non-SAMU total (n = 74)	
Time since graduation (years) (median, IQR)	5.3 (1.8-12.7)	2.3 (1.5-5.0)*	11.0 (2.4-23.2)*	5.5 (1.9-15.3)†	5.3 (1.8-10.7)†
Male sex	93 (67.9)	13 (46.4)	35 (76.1)	48 (64.9)	45 (71.4)
**Medical category/specialty**					
Generalist	43 (31.4)	12 (42.9)	8 (17.4)	20 (27.0)	23 (36.5)
Specialty	94 (68.6)	16 (57.1)	38 (82.6)	54 (73.0)	40 (63.5)
Internal medicine	40 (29.1)	9 (32.1)	18 (39.1)‡	27 (36.4)‡	13 (20.6)
Pediatrics	13 (9.4)	3 (10.7)	5 (10.8)	8 (10.8)	5 (7.9)
Surgery	10 (7.2)	1 (3.5)	4 (8.6)‡	5 (6.7)‡	5 (7.9)
Gynecology and Obstetrics	10 (7.2)	1 (3.5)	6 (13)‡	7 (9.4)‡	3 (4.7)
Cardiology	4 (2.9)	0 (0)	0 (0)	0 (0)	4 (6.3)
Family Medicine	4 (2.9)	0 (0)	0 (0)	0 (0)	4 (6.3)
Others§	16 (11.6)	2 (7.1)	8 (17.3)‡	10 (13.5)‡	6 (9.5)

SAMU: mobile emergency care service; IQR: interquartile range.

* Comparison of the time since graduation between physicians of level II hospitals and level III/IV hospitals: p ≤ 0.01;

† Comparison of the time since graduation between SAMU and non-SAMU physicians: p = 0.64;

‡ Two physicians had multiple specialties: one had two specialties (Internal Medicine and Surgery) and the other, three (Anesthesiology, Gynecology and Obstetrics, Labour Medicine). Both worked at a level III/IV hospital;

§ Others: Anesthesiology (3, 1 at SAMU and 2 at level III/IV hospital), Cardiovascular Surgery (2, at SAMU), Thoracic Surgery (2, 1 at SAMU and 1 at level III/IV hospital), Intensive Care Medicine (2, 1 at SAMU and 1 at level III/IV hospital), Neurology (1, at level II hospital), Dermatology (1, at level II hospital), Traffic Medicine (1, at SAMU), Labour Medicine (2, at level III/IV hospital), Orthopedics and Traumatology (1, at level III/IV hospital) and Psychiatry (1, at level III/IV hospital).

Most respondents showed overall dissatisfaction with the structure of care provided to cardiovascular emergencies in the region, whose median of the overall scale was 2.0 (IQR 2.0-4.0). When assessing *“overall satisfaction”*, the dissatisfaction of SAMU physicians was lower (p = 0.01). In addition, the physicians of level III/IV hospitals showed higher *“overall satisfaction”* as compared to those of level II hospitals (p ≤ 0.05) (Table 2). No statistically significant correlation was observed between professional training time and *“overall satisfaction”* [r_s_ = 0.112, p = 0.195].

**Table 2:** Comparison of the satisfaction of physicians (CARDIOSATIS-Team scale) categorized according to the type of emergency service, and result of the comparison test between groups

Domains/Itens of the scale	Overall (n = 137)	Non-SAMU (n = 74)				SAMU (n = 63)	Comparison between SAMU and non-SAMU(p-value)*
		Level II hospitals (n = 28)	Level III/IV hospitals (n = 46)	Non-SAMU total (n = 74)	Comparison between level II hospitals and level III/IV hospitals (p-value)*		
Domain 1: Satisfaction with the care provided (5 items)	2.0 (2.0-4.0)	2.0 (2.0-4.0)	2.0 (2.0-4.0)	2.0 (2.0-4.0)	0.96	2.0 (2.0-4.0)	0.05
Satisfaction with the care provided	2.0 (2.0-4.0)	4.0 (4.0-4.0)	3.5 (2.0-4.0)	4.0 (2.0-4.0)	0.38	2.0 (2.0-4.0)	0.87
Municipality's structure for diagnosis	2.0 (2.0-4.0)	2.0 (2.0-4.0)	2.0 (2.0-3.0)	2.0 (2.0-3.5)	0.49	2.0 (2.0-4.0)	0.03
Structure for managing cardiovascular diseases	2.0 (2.0-4.0)	2.0 (2.0-4.0)	2.0 (2.0-4.0)	2.0 (2.0-4.0)	0.34	2.0 (2.0-4.0)	0.59
Diagnostic accuracy	2.0 (2.0-4.0)	2.0 (2.0-2.0)	2.0 (2.0-4.0)	2.0 (2.0-4.0)	≤ 0.05	2.0 (2.0-4.0)	0.01
Technical support	5.0 (5.0-5.0)	5.0 (5.0-5.0)	5.0 (1.0-5.0)	5.0 (1.0-5.0)	0.50	5.0 (5.0-5.0)	≤ 0.01
Domain 2: Structure of care and diagnosis (6 items)	2.5 (2.0-3.5)	2.0 (2.0-2.0)	2.5 (2.0-3.5)	2.0 (2.0-3.0)	≤ 0.001	3.0 (2.0-4.0)	≤ 0.001
Medical facilities for the diagnosis of cardiovascular diseases	3.0 (2.0-4.0)	1.0 (1.0-2.0)	3.0 (2.0-4.0)	3.0 (2.0-3.0)	≤ 0.001	3.0 (2.0-4.0)	≤ 0.001
Quality of the equipment and materials	3.0 (2.0-3.0)	2.0 (2.0-2.0)	3.0 (2.0-3.0)	3.0 (2.0-3.0)	0.12	3.0 (3.0-4.0)	≤ 0.001
Technology available for diagnosis	2.0 (2.0-3.5)	2.0 (2.0-2.0)	2.0 (2.0-3.0)	2.0 (2.0-3.0)	0.66	2.0 (2.0-4.0)	≤ 0.001
Promptness in diagnosis	2.0 (2.0-3.5)	2.0 (2.0-2.0)	2.0 (2.0-4.0)	2.0 (2.0-3.0)	≤ 0.01	2.0 (2.0-4.0)	≤ 0.001
Adequacy of the service	3.0 (2.0-3.0)	2.0 (1.0-2.0)	3.0 (3.0-3.0)	3.0 (2.0-3.0)	≤ 0.001	3.0 (3.0-4.0)	≤ 0.001
Resolutivity	2.0 (2.0-4.0)	2.0 (2.0-2.0)	2.0 (2.0-4.0)	2.0 (2.0-4.0)	≤ 0.001	3.0 (2.0-4.0)	≤ 0.001
Overall scale (11 items)	2.0 (2.0-4.0)	2.0 (2.0-2.0)	3.0 (2.0-4.0)	2.0 (2.0-3.0)	≤ 0.05	2.0 (2.0-4.0)	≤ 0.001

Values expressed as median (interquartile range), except when indicated;

* Comparative analysis by use of Mann-Whitney U test.

When assessing the scale domains, slightly higher “*satisfaction with the structure of care and diagnosis”* (median 2.5, IQR 2.0-3.5) was observed as compared to *“satisfaction with the care provided*” (median 2.0, IQR 2.0-4.0). In the domain *“satisfaction with the care provided*”, a significant difference was observed between the groups regarding *technical support*, perceived as worse by the hospital physicians. In the domain *“structure of care and diagnosis”*, the satisfaction of the hospital physicians with *“medical facilities for the diagnosis of cardiovascular diseases”*, *“promptness in diagnosis”*, *“adequacy of the service”* and *“resolutivity”* was lower as compared to that of SAMU physicians. In addition, the satisfaction of physicians of level II hospitals with those same items was lower than that of physicians of level III/IV hospitals. The satisfaction with the *“technology available for diagnosis”* was lower among the hospital physicians as compared to that of SAMU physicians, but did not differ between the two subgroups of hospital physicians.

**Figure 1:**
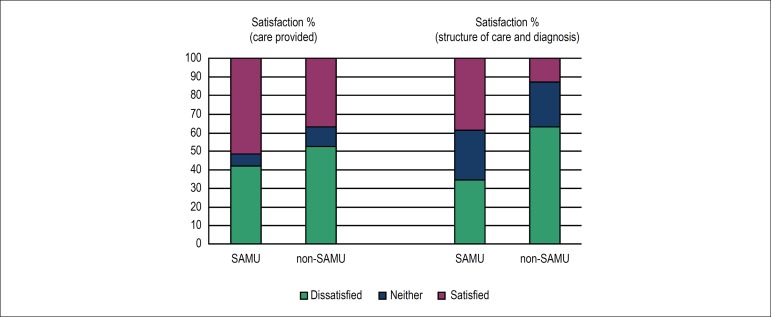
Satisfaction of physicians of the mobile emergency service (SAMU) and of hospital emergency services (non-SAMU) according to the domains of the CARDIOSATIS-Team scale.

**Figure 2:**
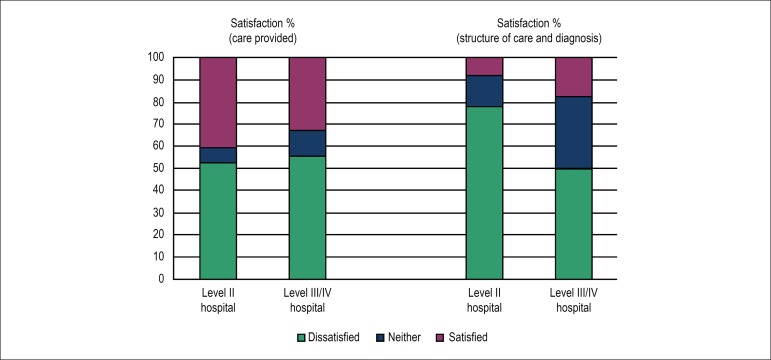
Satisfaction of physicians of level II hospitals and those of level III/IV hospitals according to the domains of the CARDIOSATIS-Team scale.

When comparing SAMU physicians with those working at hospital emergency services, the former showed a higher satisfaction level in both domains (Figure 1, Table 3). When comparing physicians working at level II hospitals with those at level III/IV hospitals, the satisfaction with the care provided was similar. However, when assessing the domain *“structure of care and diagnosis”*, the physicians working at level III/IV hospitals were more satisfied (Figure 2, Table 4).

**Table 3:** Description of the satisfaction level of physicians of the mobile emergency care service (SAMU) and of hospitals (non-SAMU) according to the CARDIOSATIS-Team scale

Domains/Itens of the scale	SAMU (n = 63)			Non-SAMU (n = 74)		
	Dissatisfied (1-2)	Neither (3)	Satisfied (4-5)	Dissatisfied (1-2)	Neither (3)	Satisfied (4-5)
**Domain 1: Satisfaction with the care provided (5 items)**						
Satisfaction with the care provided	34 (54.0)	1 (1.6)	28 (44.4)	37 (50.0)	7 (9.5)	30 (40.5)
Municipality's structure for diagnosis	29 (46.0)	3 (4.8)	31 (49.2)	49 (66.2)	8 (10.8)	17 (23.0)
Structure for managing cardiovascular diseases	33 (52.4)	7 (11.1)	22 (34.9)	46 (62.2)	7 (9.5)	21 (28.4)
Diagnostic accuracy	25 (39.7)	10 (15.9)	28 (44.4)	44 (59.5)	14 (18.9)	13 (17.6)
Technical support	9 (14.3)	-	52 (82.5)	11 (14.9)	-	52 (70.3)
**Domain 2: Structure of care and diagnosis (6 items)**						
Medical facilities for the diagnosis of cardiovascular diseases	20 (31.8)	14 (22.2)	29 (46.0)	42 (56.8)	18 (24.3)	13 (17.6)
Quality of the equipment and materials	11 (17.5)	31 (49.2)	21 (33.3)	34 (46.0)	34 (46.0)	5 (6.8)
Technology available for diagnosis	34 (54.0)	9 (14.3)	20 (31.8)	58 (78.4)	7 (9.5)	9 (12.2)
Promptness in diagnosis	30 (47.6)	6 (9.5)	27 (42.9)	52 (70.3)	11 (14.9)	11 (14.9)
Adequacy of the service	10 (15.9)	30 (47.6)	23 (36.5)	40 (54.1)	24 (32.4)	7 (9.5)
Resolutivity	26 (41.3)	11 (17.5)	26 (41.3)	49 (66.2)	11 (14.9)	12 (16.2)

Values expressed as n (%).

**Table 4:** Description of the satisfaction level of physicians of level II hospitals and level III/IV hospitals according to the CARDIOSATIS-Team scale

Domains/Itens of the scale	Level II hospitals (n = 28)			Level III/IV hospitals (n = 46)		
	Dissatisfied (1-2)	Neither (3)	Satisfied (4-5)	Dissatisfied (1-2)	Neither (3)	Satisfied (4-5)
**Domain 1: Satisfaction with the care provided (5 items)**						
Satisfaction with the care provided	13 (46.4)	1 (3.6)	14 (50.0)	24 (52.2)	6 (13.0)	16 (34.8)
Municipality's structure for diagnosis	18 (64.3)	2 (7.1)	8 (28.6)	31 (67.4)	6 (13.0)	9 (19.6)
Structure for managing cardiovascular diseases	16 (57.1)	2 (7.1)	10 (35.7)	30 (65.2)	5 (10.9)	11 (23.9)
Diagnostic accuracy	21 (75.0)	4 (14.3)	2 (7.1)	23 (50.0)	10 (21.7)	11 (23.9)
Technical support	9 (32.1)	-	19 (67.9)	18 (39.1)	-	27 (58.7)
**Domain 2: Structure of care and diagnosis (6 items)**						
Medical facilities for the diagnosis of cardiovascular diseases	24 (85.7)	1 (3.6)	3 (10.7)	18 (39.1)	17 (37.0)	10 (21.7)
Quality of the equipment and materials	15 (53.6)	12 (42.9)	1 (3.6)	19 (41.3)	22 (47.8)	4 (8.7)
Technology available for diagnosis	21 (75.0)	3 (10.7)	4 (14.3)	37 (80.4)	4 (8.7)	5 (10.9)
Promptness in diagnosis	23 (82.1)	3 (10.7)	2 (7.1)	29 (63.0)	8 (17.4)	9 (19.6)
Adequacy of the service	23 (82.1)	3 (10.7)	1 (3.6)	17 (37.0)	21 (45.7)	6 (13.0)
Resolutivity	23 (82.1)	2 (7.1)	2 (7.1)	26 (56.5)	9 (19.6)	10 (21.7)

Values expressed as n (%).

## Discussion

This study involved physicians working in the public emergency services of the Northern Region of Minas Gerais (SAMU and emergency units of hospitals of different levels of complexity). Most of them had a short time since graduation, were male and specialists (68.6%). In addition, most of them expressed overall dissatisfaction with the care provided to cardiovascular diseases. SAMU physicians expressed higher level of satisfaction with the structure of cardiovascular care as compared to those working at the regional hospitals. In both groups, most physicians were satisfied with the “*technical support”* for the management of a patient, while most SAMU physicians were dissatisfied with the “*care provided”* and “*technology available for diagnosis”* (54% for both), and most hospital physicians were dissatisfied with the “*technology available for diagnosis”* (78.4%) and “*promptness in diagnosis”* (70.3%).

The health system of the Northern Region of Minas Gerais is a hierarchical regional emergency care network.^13^ Oliveira et al.^18^ have reported that the health system would be better considered as a circuit with multiple entry points, in which there is a more suitable place for each patient regarding the required type of care. When referring a patient to an emergency service, SAMU regulatory center physicians should always consider the best option regarding the resources available, the location of the teams and proximity.^19^ In the Northern Region of Minas Gerais, as SAMU is regionalized, the number of advanced ambulances is limited. Because that number is calculated based only on a population criterion, ignoring the long distances, more often than not the closest advanced support is an emergency center of a regional hospital, independently of the severity of the patient’s condition or even of the technical skills of the team.

In the present study, more than 50% of the hospital physicians expressed dissatisfaction with 9 of the 11 items. Those professionals highlighted the inadequacy of the emergency units, which involves the quality of equipment and materials, in addition to the municipalities’ limited structure for diagnosis, which reflects on the overall quality of the cardiovascular care provided.

It is worth noting that the physicians of level II hospitals expressed more dissatisfaction than those of level III/IV hospitals regarding the *structure of care and diagnosis*, such as the *medical facilities for the diagnosis of cardiovascular diseases*, *promptness in diagnosis, adequacy of the service,* and *resolutivity*, even if, by definition, the structure of a level II hospital is better than that of level III/IV hospitals. The number of dissatisfied physicians was higher among those of level II hospitals regarding the domains *“medical facilities for the diagnosis of cardiovascular diseases”*, *“promptness in diagnosis”*, *“adequacy of the service”* and *“resolutivity”*, in which a lower level of dissatisfaction would be expected among physicians of level II hospitals than the ones of level III/IV hospitals. It might be due to the higher expectations of those professionals, because satisfaction is known to relate to both adequacy of the services and individuals’ expectations regarding quality care.^16^^,^^20^

It is worth noting the high number of physicians without medical residency (31.4%) – generalists – or from medical specialties without specific training in adult cardiovascular emergency (pediatrics and gynecology). The findings of the present study show the importance of promoting continuous education programs in the region to improve the skills of the physicians working at cardiovascular emergency services. The highest satisfaction level with “*technical support”* is positive in that context. In addition, such findings emphasize the need for training in emergency medicine in the medical curriculum. In Brazil, physicians graduate without the necessary work experience in the emergency setting. That has been recognized by the *Associação Brasileira de Educação Médica* (Brazilian Association of Medical Education), which, nevertheless, reports that “most newly graduated physicians end up on work shifts at emergency units or pre-hospital care units”, but the “*Diretrizes Curriculares Nacionais”* (National Curriculum Guidelines) do not value that area of medical practice.^21^

The previous “*Diretrizes Curriculares Nacionais”* (National Curriculum Guidelines) for medical education did not include emergency medicine in the required disciplines of the medical internship.^22^ The current ones require that at least 30% of the hours of the medical internship be spent in Primary Care and Emergency Care of the Brazilian Unified Health System (SUS), “respecting the minimum of two years of internship”.^23^ However, the number of hours dedicated to emergency education is still limited in most medical schools in Brazil,^24^ which tends to aggravate with the ever-increasing number of medical schools and the scarcity of practice scenarios.

In 2015, emergency medicine was recognized as a medical specialty by the “*Conselho Federal de Medicina*” (Brazilian Federal Council of Medicine), the “*Conselho Nacional de Residência Médica*” (National Council of Medical Residency) and the “*Associação Brasileira de Educação Médica*” (Brazilian Association of Medical Education). Although that qualification in emergency care was being structured during the time this study was being performed, so far there is no official medical education program for pre-hospital care.

Currently, emergency services face great challenges in several realms: scarcity of skilled labor, overcrowded facilities, low quality of care provided to those who most need it high turnover of professionals, and exposure of professionals to risks due to the growth of violence in large cities.^19^ Several studies have assessed the organization of emergency services, but data analyzing those professionals’ satisfaction are scarce. Another study assessing the physicians’ satisfaction with the structure of cardiovascular care has been conducted in the same region, but with professionals working in primary healthcare before and after the implementation of a Telehealth system in cardiology.^16^

Studies have investigated the burnout of physicians. Its frequency among emergency professionals is alarming.^25^ Work dissatisfaction is one of the burnout-related factors reported. A study of 771 North American emergency physicians has observed that those reporting stress and burnout as severe problems expressed lower levels of satisfaction with their careers.^26^ Another study of 193 North American emergency physicians members of the American College of Emergency Physicians, has reported that dissatisfaction related to clinical autonomy, to challenges in the emergency medicine practice and to stress were significantly associated with high levels of burnout.^27^ Our study was not aimed at specifically investigating burnout in that population, but the high dissatisfaction level found indicates the need for specific assessments.

This study has limitations inherent in its cross-sectional design, preventing inference of causality. Other factors might have affected the physician’s satisfaction, such as professional acknowledgement, changes in salary and better working conditions, which were not directly measured in this investigation.^16^

The results of the present study are important because they enable managers of pre-hospital and hospital emergency services to reflect, aiming at qualifying the care provided. Negative changes in the mental state of emergency professionals have a adverse impact on their professional performance.^28^ The satisfaction with the structure of cardiovascular care of the SAMU or hospital physicians found in this study was extremely important to delineate and implement the AMI system of care. The operational flow was discussed with the managers, the tele-electrocardiogram was installed in the ambulances and the thrombolytic was acquired,^11^ however, without the adherence and motivation of the physicians and nurses at the emergency services, the AMI system of care would be doomed to failure. This is a pioneer study in the assessment of the baseline for the implementation of the AMI system of care in Brazil, which can be a model for future implementations. Additionally the results can help the assessment of the quality of the care provided and the planning of training programs, guiding the definition of priorities, mainly for services that provide care for cardiovascular diseases.^16^

## Conclusion

This study showed the overall dissatisfaction of emergency physicians in the Northern Region of Minas Gerais with the structure of care provided for cardiovascular emergencies. Most physicians expressed dissatisfaction with the care provided, the structure for managing cardiovascular diseases and the technology available for diagnosis. The dissatisfaction of SAMU physicians was lower as compared to that of the emergency physicians at the regional hospitals, and the dissatisfaction of physicians of level III/IV hospitals was lower as compared to that of physicians of level II hospitals.
